# Struggling with the governance of interprofessional elderly care in mandated collaboratives: a qualitative study

**DOI:** 10.1186/s12913-023-09026-1

**Published:** 2023-01-11

**Authors:** Rabab Chrifou, Hanna Stalenhoef, Kor Grit, Jozé Braspenning

**Affiliations:** 1 Radboud University Medical Centre, Radboud Institute for Health Sciences, Scientific Centre for Quality of Healthcare (IQ Healthcare), Nijmegen, The Netherlands; 2 Faculty of Medicine and Health Sciences, Department of Public Health and Primary Care, Ghent University, Ghent, Belgium; 3 Erasmus School of Health Policy & Management (ESHPM), Erasmus University, Rotterdam, The Netherlands

**Keywords:** Interorganisational collaboration, Mandated collaboratives, Interprofessional functioning, Governance, Integrated care, Elderly care, Qualitative research

## Abstract

**Background:**

Governing interprofessional elderly care requires the commitment of many different organisations connected in mandated collaboratives. Research over a decade ago showed that the governance relied on clan-based mechanisms, while lacking formal rules and incentives for collaborations. Awareness and reflection were seen as first steps towards progression. We aim to identify critical governance features of contemporary mandated collaboratives by discussing cases introduced by the healthcare professionals and managers themselves.

**Methods:**

Semi-structured interviews (*n* = 24) with two regional mandated collaboratives took place from November 2019 to November 2020 in the Netherlands to learn more about critical governance features. The interviews were thematically analysed by the project team (authors) to synthesise the results and were subsequently validated during a focus group.

**Results:**

Critical governance features of interorganisational activities in mandated collaboratives include the gradual formulation of shared vision and clear client-centred goals, building trust and acquaintanceship for the advancement of an open collaborative culture, establishing a non-extreme formalised governance structure through leadership, mutual trust and innovation support and facilitating information exchange and formalisation tools for optimal elderly care.

**Conclusion:**

Trust and leadership form the backbone of interorganisational functioning. Interorganisational functioning should be seen in light of their national embedment and resources that are (being made) available, which makes them susceptible to constant change as they struggle with balancing between critical features in a fluid and intermingled governance context. The identified critical features of (contemporary) mandated collaboratives may aid in assessing and improving interprofessional functioning within integrated elderly care. International debate on governance expectations of mandated collaboratives may further contribute to sharpening the roles of both managers and healthcare professionals.

## Introduction

Healthcare for elderly with chronic and disabling conditions has become more complex and integrated care is believed to improve coordination, efficiency and quality outcomes of service delivery [1]. Review studies on the effects of integrated care conclude that there is a lack of consistent and reproducible evidence, probably due to the way integrated care is defined and conceptualized [2, 3]. After all, integrated care is not an empirical phenomenon in itself, but it covers a multitude of topics, goals and strategies in which teamwork of autonomous professionals plays a key role at both care and organizational levels [2, 4]. A review suggested that less is known about the critical organisational factors that can improve interprofessional functioning in integrated care and, as such, the patient outcomes [5]. The organizational level concerns the various organizations involved in elderly care, often linked in so-called mandated collaboratives [6]. The organizations involved are represented in these mandated collaboratives on a local or regional level to govern innovative elderly care. However, the organizations and persons in the mandated collaborative may differ per project. The focus of the paper is on critical *inter*organisational activities of the mandated collaboratives to govern interprofessional functioning in elderly care.

About 10 to 15 years ago, research was started into the governance of mandated collaboratives in healthcare. It was shown that mandated collaboration requires the mobilisation of multiple activities, as the collaboration is paradoxical in nature combining competition and cooperation, and autonomy and interdependence [6]. Based on their empirical data, the governance was described as relying on clan-based mechanisms. The assumption is that mandated collaboratives function because they are a group of people united by previous joint activities. Critical features that may affect this kind of governing include leadership, service structure, culture, language, resources, credibility, shared values, openness, integrity, internalisation and accountability [6–12]. These studies are based on normative theories and meta level examples [8, 10, 11], but also empirical findings from healthcare professionals and managers [6, 7, 9, 12]. Furthermore, it is stated that the activities of the collaboratives occur in a constantly dynamic environment with varying spokespersons and changing policies. Some research indicates that governance maturity is reached in a fully formalised structure [8]. In practice, some of these collaboratives have reached top-down a more formalised structure [10], while others believe in looser structures such as collaborative platforms [11].

In the Netherlands, a national program of integrated elderly care was started in 2008 [www.zonmw.nl]. A recent report shows that the governance in mandated collaboratives is still taking shape [13]. The aim of our paper is to empirically identify the governance features of contemporary mandated collaboratives for integrated elderly care in the Dutch setting.

The research question is: What are current critical features of governing interprofessional elderly care according to healthcare professionals and managers engaged in mandating collaboratives?

The results may also add value to other settings or populations requiring integrated care, such as people with chronic mental problems or children with serious chronic diseases.

## Methods

### Study design

A qualitative study using semi-structured interviews and a validating focus group was performed to identify critical governance features in two regions. The study was designed and reported according to the consolidated criteria for reporting qualitative research (COREQ) [14]. Ethical approval for the study was given by the Erasmus University Ethical Review Board.

### Setting

The study is performed in the Netherlands. To describe the setting, we studied a recent Dutch report on the governance of interprofessional elderly care [13] and conducted semi-structured interviews (*n* = 7) with healthcare professionals and their managers. This resulted in the background information of the setting as provided in Table 1.

**Table 1:** Integrated elderly care in the Netherlands

• Integrated elderly care is described in national elderly care programmes, initiated by primary care. In the Netherlands approximately 20% of the population is older than 65 years.
• Involved healthcare professionals are providing general practice care (gatekeeper), allied care (including district nursing, physio- and occupational therapy), home care services, social work, specialist (elderly) care. Day care is delivered by social care or specialist care.
• Involved organisations include primary care groups, care organisations (district nursing, home care services) and often additionally social work, municipalities and/or hospitals.
• Financers are health insurers, municipalities and government.
• Formalisation is still being developed but resembles *mandated collaboratives* [6]. Participants of such collaboratives are managers and coordinators of the organisations. In the Netherlands, there can be over 50 health organisations involved in elderly care in one region, including regional patient organisations. Managerial delegates from the key organisations meet at ‘round tables’ to set the regional agenda and discuss ongoing projects. It can be agreed that a certain organisation represents the others working in the same sector. Although there are some initiatives that strive for more formalisation, it is also proclaimed that a higher level of formalisation is not pursued to ensure flexibility over time.

The situation in region A and B is similar to that described in Table 1. Region A was located in the east of the Netherlands and region B in the north-west of the Netherlands. In the collaboratives of both regions certain key organisations were naturally involved and complemented by other organizations depending on the project. The healthcare professionals and managers involved met each other more or less regularly on collaborative platforms to support integrated elderly care. Citizens, in our case older people, were often represented in panels within wider organisations and engaged in projects, but not structurally in the mandated collaboratives.

### Participants

Participants were included if they met the criterion of being directly or indirectly active in one of the organizations in the region involved in elderly care. The voluntary participants were identified using a combination of purposive and snowball sampling. For the regions, we purposively searched the Internet for names of healthcare professionals and managers of key organizations in the region. In addition, we asked the interviewees who we should definitely speak to and whom we could invite to participate in the focus group. We explained that we would like to interview people who worked at the management level from the key organizations participating in the mandated collaboratives. We aimed for diversity in organization and function. Table 2 describes the organisations and the function of the participants. In total, 24 respondents (four double interviews, see merged cells) participated in the interviews, 12 in each region (A and B). Five persons joined the focus group. The age of the participants varied approximately between 30 and 70 years, 45% were female. The majority of participants had more than five years of working experience in in the health sector.

**Table 2:** Organisation and function of participants

Region A: interview participants		Region B: interview participants	
Organisation	Function	Organisation	Function
Primary care collaborative	Coordinator	Primary care collaborative	Chair, GP
Dementia collaborative	Director	District nursing, home services	Director
Home services	Director	Primary care group	Staff
Home services	Project manager	Primary care group	Policy officer, GP
Primary care group	GP	Social welfare	Manager
Specialist elderly care	Director	Municipality	Policy advisor
Social welfare	Manager	Municipality	Policy advisor
Municipality	Policy advisor	Hospital	Policy advisor
Municipality	Policy advisor	Patient elderly organisation	Coordinator
Hospital	Policy advisor	Health insurer	Innovation manager
Patient elderly organisation	Chair	Health insurer	Local manager
Patient elderly organisation	Assistant	Network elderly care	Coordinator
**Region A: focus group participants**		**Region B: focus group participants**	
Specialist elderly care	Director	Elderly patient organisation	Chair
Collaborative platform	Coordinator	Social welfare	Director
Primary care group	Coordinator		

### Data collection

The one-hour interviews with the collaborative partners of the two regions were held during March–June 2020, and the focus group (two hours) was on November 11, 2020. Due to travel restrictions, as a result of the COVID-19 outbreak, the interviews and focus group in 2020 were held online. All interviews were recorded with consent. The interviews were performed mainly by RC or HS and sometimes accompanied by JB and KG, respectively. JB and KG are experts in the field and have pre-existing knowledge on the subject.

The interview guide for exploration of the setting and the features of the functioning of the collaboratives was based on the typology of D’Amour and colleagues [12]. The model was chosen due to its representativity of previously described features for interprofessional collaboration [6–12]. Some words in the description of the typology were modified to gain more focus on the *inter*organisational level, see Table 3. The interview started with a description of the participants in the collaborative and their activities. The main findings from the semi-structured interviews were validated in a focus group by asking questions on recognizability and necessary additions [15].

**Table 3:** Typology of (interorganisational) collaboration based on the model of D’Amour and colleagues [12]

**Theme**	**Subtheme**	**Short description**
**Shared goals and vision**	Client-centred goals	Identifying and sharing common goals is essential for a collaborative.
	Negotiation of interest	Complex structures of different interests are a risk to loss of focus on client-centred collaboration.
**Internalisation**	Mutual acquaintanceship	Knowing each other personally means knowing each other’s values, level of competence and becoming acquainted with the professionalism of each agency.
	Trust	Collaboration is possible having trust in each other’s competencies and ability to assume responsibilities.
**Governance**	Centrality	Centrality refers to the existence of clear and explicit direction that is meant to guide the collaboration.
	Leadership	Local leadership is necessary for the development of interorganisational collaboration. If leadership is related to a position, power should not be concentrated in the hands of a single agency; all agencies must be able to have their opinions heard and to participate in decision-making.
	Support for innovation	New activities and responsibilities between the organisations must be developed and implemented. This is a learning process.
	Connectivity	Connectivity refers to the fact that the organisations are interconnected, that there are places for discussion and for constructing bonds between them. It solves coordination problems and enables adjustments.
**Formalisation**	Tools	Tangible products that serve as facilitators in interorganisational collaboration, such as agreements, protocols and information systems. Collaboration is less influenced by the degree of formalisation than by the consensus that emerges around formalisation mechanisms and the specific rules that are implemented.
	Information exchange	The exchange of information refers to the existence and appropriate use of an information infrastructure to allow for rapid and complete exchanges of information between professionals and organisations. Feedback is an important aspect of establishing relationships of trust.

After two-third of the interviews, data saturation was discussed and confirmed within the research team. Due to function and region diversity, it was decided to add extra interviews.

### Data synthesis

Interview and focus group data were transcribed and analysed using a thematic analysis [16] based on the themes extracted from Table 3. Computer assisted analysis software (Atlas.ti) was used to accelerate the coding process. The categorisation of the information was continuously and thoroughly discussed by the research team (RC, HS, KG, JB) to ensure a robust description of the perspectives and experiences of the involved stakeholders. The information derived from the focus group served as a validation for the interviews and as a completion of the analysis. In reporting the quotes, the organisation and function of the speaker are made clear.

## Results

### Shared vison and goals: client-centred goals and the negotiation of interest

Respondents emphasised that shared vision and goals are needed to consolidate collaboration. General descriptions of shared vision are used as ‘putting the client at the centre’, ‘the right care in the right place’ or ‘interprofessional collaboration’. According to the respondents, more concrete goals were formulated in the separate, theme-based projects. Refinement along the way across healthcare organisations was preferred over starting a long discussion beforehand:

The first thing we started doing four years ago was to connect with those other home care and nursing organisations. And to see whether it is possible to develop one vision together. That is quite complicated, by the way. Well, that takes time. And I have to say, step by step it is going in a certain direction. (Director specialist elderly care)

Implementing these goals means that all organisations involved should benefit from the collaboration. Interests of the different organisations should be considered or negotiated to achieve a sustainable collaborative:

I think there is still a lot of work to be done when it comes to interests. If you want to build a very strong network, you must of course first share the same ambitions, but then those interests really must also be on the table: why are you doing this? Why is this important to you? What opportunities do you see and what threats? And those interests are far from being on the table at the moment. (Policy advisor hospital)

One example of underlying interests which may hinder collaboration in the long run is losing market shares. Allocated market shares from municipalities and health insurers cause division between organisations in the region in terms of acting power and control, which potentially thwarts the formulation of shared goals and vision of organisations involved in the first place:

And I have to say that the current model, in which health insurers buy in every year and influence the extent to which an organisation can provide care, is incredibly frustrating, and also it does not help with this development (of network collaboration). So, I suffer from that too. And we all suffer from that. Because on the one hand we have these great ambitions and we all want to, but on the other hand we also negotiate with those insurers every year and we try to get the biggest piece of the pie. (Director specialist elderly care)

By making the aims of the collaborative more explicit at an early stage, discussions on conflicting interests are required, which facilitates the implementation of project goals.

### Internalisation: mutual acquaintanceship and trust

Acting in a relatively small area is beneficial for communicative purposes and building on acquaintanceship and trust. In general, it seems that more effort is needed to build trust and be acquainted with each other at an interorganisational level as opposed to the interprofessional level, especially when there is no history of collaboration. Internalisation takes more effort if more organisations are involved. A trajectory is needed for collaborators to know each other professionally and personally and to build trust. As illustrated by the quote below, a proactive attitude from collaborators is needed:

What we have to do, if we want true network collaboration, is more than just calling or emailing. We really have to invest in relationships with each other and create the same picture. (Coordinator patient elderly organisation)

Several managers emphasised the importance of creating a culture in which professionals understand their tasks and where openness and trust are the norm. According to them, trusting each other’s knowledge and capabilities has grown over the past years. However, the development of trusting relationships is sometimes hindered by internal agreements within one of the involved organisations or by competing for finance from healthcare insurers or municipalities:

Lack of collaboration is ultimately quite frustrating. And it still gives rise to a kind of basic mistrust: do you still talk to that insurer? And sometimes we just compete (with similar organisations) and try to attract future growth. Those kinds of classic patterns still reappear. That really disturbs me, but it does happen. And I just go along with it. Let that be clear too. But of course, it is just bad for network development. So, it is also constantly navigating between trust, building a relationship… (Director specialist elderly care).

Common language and knowing each other’s tasks were described as facilitators for internalisation, and besides time, require curiosity as possibilities to meet had to be created:

It’s not that you encounter each other in the corridors. You only meet each other if you really seek to meet. And then there must be lines. Then I come back to my first comment about synergy and curiosity. Those are conditions. And you just have to have time for that again. (Director social welfare)

### Governance: centrality, leadership, support for innovation, connectivity

#### Centrality

The collaboratives started mostly from one or two joint projects to implement parts of the Dutch national programme on integrated elderly care. Harmonising tasks, responsibilities and finances seem to be the main drivers for the interorganisational collaboration. Governance structures to guide interorganisational collaboration differ in their degree of formalisation. Some stated that it suffices to have a couple of enthusiastic members who lead and organise interorganisational meetings, while others stressed the importance of formalisation to mandate projects initiatives. At least it seems common to conclude on covenants between the key organisations within the collaboratives, that is, general practices represented by their regional association, organisations that provide district nursing and home care services, and social work organisations.

Agreements are made about representatives. In practice this means that mainly the larger organisations participate; they have more resources (financial, capacity, knowledge) to be able to invest in collaboration. Little energy is put into defining the region or catchment area, because the various organisations involved each serve different regions, and for the necessary care it is especially important that the partners (also outside regional borders) know each other.

Respondents agreed that the collaborative body should not be authoritative with an imperative hierarchical structure, rather it should function as a ‘vehicle’ or facilitator to improve and accelerate collaboration between organisations:

In my opinion, structuring, formalising, is not always a mandatory condition for me to be able to talk about a well-functioning collaborative. (Chair elderly patient organisation)

Collaboration on an interorganisational level is predominantly based on projects, as these initiatives need reconciliation and coordination. Patient organisations are often present at the projects, but they hardly participate in the regular meetings with the key organisations. The same applies for municipalities and health insurers. However, the latter two are decisive on the available budget and as such, influence the activities:

The municipality determines the scope within an area. Through conversations and contract meetings we tried to facilitate collaboration within the area. For years we have been looking for the key to how collaboration within care collaboratives can be improved. (Policy advisor municipality)

Nevertheless, a limited form of formalisation is being pursued. This could include appointing a coordinator and limited support staff to prepare for a meeting. This coordination is intended to maintain an overview of several regional and national projects and can support the sustainability of new initiatives.

#### Leadership

The importance of leadership was underlined, defined as being able to propagate the goal, being trustworthy and communicative, being able to stimulate and encourage others and being positive, inspirational, realistic and decisive:

It is not clear whether a structure can change the underlying tension, especially leadership can play a role in this. For example, making yourself vulnerable is what the director does, you notice that within the organisation, you can say anything to that person. (Manager social welfare)

Collective leadership – embodied by multiple parties – was stressed as creating impact and power in the region:

When there is no “click” between people, when there is no trust between people, then you will not get any further (in collaboration). It also doesn’t help when it’s up to someone who can’t take it further. I am powerful when I work together as a team, and not just as one party, when you are with multiple parties, then you have more coverage in the region. (Director district nursing, home services)

Some organisations mentioned that they have a historical advantage of being in the lead in a certain region because of their volume, proactive attitude or position. These ‘key’ organisations felt they had a good position in the region and felt a certain responsibility to initiate and carry collaborative initiatives in the region. One of the respondents spoke of a kind of legacy:

And there is also a cultural aspect to it. That we have actually been initiators of new developments in the region for many years… We now also see that we are very much granted that regional pioneering role. (Project manager home services)

#### Support for innovation

The extent to which training and academic workspaces are offered by the collaboratives to improve integrated elderly care and thus interprofessional collaboration is limited. Respondents reacted in a surprised manner as the interviewer suggested supporting a common educational programme for the healthcare professionals as well as the managers, but the idea was embraced by both. More in general support for innovation was evident from the various projects that were set up and carried out. Project finance and implementation fidelity were seen as barriers especially in the current context of market forces in healthcare. This made the support of innovation challenging:

In the elderly care, financial frameworks are created… you can experiment more, but there is tension between the professional expertise and (profitable) financing. There is still a challenge there. (Policy officer primary care group)

#### Connectivity

The awareness of healthcare professionals of being part of a collaborative was present and growing gradually. Developing mutual trust and getting acquainted within collaboratives is a preliminary stage for creating strong ties. Being connected at an interorganisational level adds an extra dimension to the collaboration. However, organising connectivity across multiple organisations is difficult and not always appreciated, partly due to the busy schedule of directors:

The board meets very frequently for all kinds of projects, initiatives, etc. There is an enormous meeting culture that needs to be reduced. It is more efficient if there can be a joint agenda and administrative consultation in which decisions are made, direction is given if something escalates and to facilitate collaboration. (Policy advisor hospital)

The joint agenda needs to be launched by the collaborative members within the collaborating organisations. The connectivity demands information exchange within and between the organisations to support the (change) management. Meetings to discuss progress are a manner of safeguarding these responsibilities. Unfortunately, there is little continuity among directors and managers, which impedes the development and advancement of innovations.

### Formalisation: tools and information exchange

Formalisation includes the establishment of responsibilities and rules through agreements and protocols and ensuring information exchange by setting up infrastructures to do so. It was indicated that to have formalisation tools and to exchange information in order to establish optimal patient care were crucial. According to respondents, intelligent electronic systems for exchanging information between healthcare professionals remain an obstacle for optimal performance. Furthermore, legal privacy restrictions hinder the sharing of patient information between professionals, but thorough discussions on who is authorised for which client information (medical, social) should also be organised.

In general, little to no attention was paid during meetings to the equipment of the healthcare professionals or how they became motivated to perform in a collaborative. The supervisory boards of the separate organisations often kept an eye on the quality of services and collaboration with partners. However, respondents acknowledged that more time and action were needed to monitor the client outcomes at an interorganisational level as well as the manner of cooperation itself:

We still have a long way to go. We do not yet have that much experience in working in collaboratives. This collaborative functions well, there is a lot of energy in it. There is good administrative consultation. A collaborative agreement is included. We have formulated quality criteria. And there we are about now. (Director specialist elderly care)

## Discussion

### Principal findings

The current study aimed to identify the current critical features of governing interprofessional elderly care according to healthcare professionals and managers engaged in mandating collaboratives. Our findings showed that, according to healthcare professionals and managers from the organizations engaged in current Dutch mandated collaboratives, critical governance features are: client-centred goals, negotiation of interests, mutual acquaintanceship, trust, centrality, leadership, support for innovation, connectivity, formalisation tools and information change. The degree of formalisation is kept low and the guiding principles of governance are trust and leadership. This approach implied that little attention is paid to the negotiation of interests, which makes it more difficult to implement the innovations in a sustainable manner. Moreover, the necessary resources are often lacking for the desired innovations. The fact that the mandated collaboratives are to a limited extent linked to the political or system level makes longer-term decisions and structural financing more complicated.

### Scientific and practical implications

The subthemes defined in D’Amour and colleagues’ model of interprofessional collaboration [12] can be applied to describe critical features for *inter*organisational collaboration as well. The critical features are known and can be interpreted as criteria for a qualitative functioning of collaboratives. Moving forward requires a fundamental debate about governance expectations and monitoring of the critical features. For practical reason we suggest to focus the monitoring on five interconnected issues, including: (1) the degree of formalisation, (2) trust and leadership, (3) negotiating interests, (4) available resources, and (5) connectivity between care, organisation and system levels. We will discuss each of these critical features, and formulate points of attention for the governance.

- *Formalisation* within a collaborative is seen as a way of cultivating trust by contractually defining tasks and accountability [17]. Therefore, formalisation can likely contribute to trust, another critical feature in interorganisational collaboration. On the other hand, the pragmatic approach of initiating and completing projects can also be seen as a way to enhance sustainability in the collaboration and may constitute another path for trust-building [18]. In the latter case, the role of formalisation is taken over by trust. However, the expressed high turnover of the people mandated by their organisation to join the collaborative can be a factor that hinders this kind of reinforcing sustainability. Understanding these mechanisms and being able to apply them in a specific setting can probably support a collaborative in making a more conscious choice for an appropriate degree of formalization.
- *Trust and leadership* are seen as drivers for successful governance of the interorganisational collaboration in healthcare [19, 20]. Developing mutual trust facilitates the construction of collaboratives [12, 21]. Previous joint activities act as enablers of trust for the collaborative. However, in order to maintain trust, it must be enforced through mutual successes and therefore, the results established must be realistic and agreed upon by all parties, which in turn strengthens the trust in the parties; otherwise, trust is counterproductive [17]. This maintenance aspect of trust received little attention from the collaboratives in our study. Trust and leadership appear to be a double unit, but leadership emerges in the interviews as the golden bullet for governance. Although a high turnover of the managers who participated in the collaboratives makes it vulnerable to attach leadership to one person, especially when their role and tasks are not formalised.
- *Negotiating interests* took place within innovative projects. That does not seem to be very efficient, since differences of interests – based on competing for market share or financing of activities – have to be discussed again and again, presumably with a different composition of managers present. As such, it can become a barrier to a sustainable implementation of the project results [22]. Negotiating and exploring interests of each party within the collaborative allows for more effective use of shared space and value creation for all parties involved [23]. Healthcare professionals and citizens who do not participate in negotiating interests run the risk that private interests will emerge and that there will be room for opportunistic behaviour and a shift-away from client-centred collaboration [12]. By allowing the interests to negotiate around the table, the realization of shared goals within mandated collaboratives can be safeguarded.
- *Available resources* are crucial in enabling healthcare professionals to function in mandated collaboratives, the facilitation of resources has proven to be a persistent obstacle. With regard to resources and the functioning of collaboratives, a certain paradox was identified, namely that ‘on the one hand integrated care is stimulated, but at the same time competition is stimulated and new financial structures do not facilitate integrated care’ [24]. Strengthening collaborative ties through resources should therefore be facilitated by intensifying the connection between care, organizational and system levels.
- *Three-fold connectivity* which means that interorganisational activities should not only align with the individual organisations and as such to the healthcare professionals, but also with the national policy makers (national elderly care programme, regulation of the healthcare budget and the professionals’ capacity). Working on interconnectivity further draws attention to the resources needed to shape interorganisational collaboration, such as education and finance [25].

Our study implies that the governance by collaboratives requires a shift from passive to active recognition and action appropriate to the complexity of collaborative functioning [26]. Since both healthcare professionals and managers are believed to complement each other in developing and sustaining mandated collaboratives [27], both are expected to contribute with respect to their autonomy and responsibility. Our findings suggest that healthcare professionals are still not properly supported and prepared to participate in mandated collaboratives, leaving professionals at risk of ‘overwork’: investing disproportionately in project initiatives while not being compensated by organizations or governments.

The identified five critical features could form the prelude to a national monitoring program on the functioning of the governance of mandated collaboratives. Attention should be paid to the characteristics of organisations participating in the mandated collaboratives, as this shapes the scope of mandated collaboratives and with that interprofessional functioning. Moreover, since the involvement of patient organizations is not self-evident, we formulate this as a point for improvement in current governance structures. Sharing best practices, struggles and insights on participating in mandated collaboratives has received insufficient attention to date, despite being a powerful tool for refining and defining collaborative functioning, raising awareness and cultivating reflection [26]. By acknowledging what to expect in terms of task management, resources and formalisation, both managers and healthcare professionals can prepare their roles accordingly [21].

### Strengths and limitations

A strength of this study is the robust interview guide based on the model of D’Amour and colleagues [12]. Although the model was initially drafted to explore interprofessional functioning, the four general themes proved solid enough to explore the specific critical features for interorganisational collaboration. Another strong point is the diversity of participants from the different organisations included. This resulted in a rich overview of cases and perspectives on the dynamics of interorganisational collaboration in practice. A focus group ensured the robustness of the information. A limitation can be the selection of the regions. We searched for collaboratives that were beyond their early stages and had been working together for a few years. Functioning characteristics had then crystallized somewhat.

## Conclusion

Trust and leadership form the backbone of interorganisational functioning as our findings suggest that the degree of formalisation is less important as long as trust and leadership can be built. Interorganisational functioning should be seen in light of their national embedment and resources that are (being made) available, which makes them susceptible to constant change as they struggle with balancing between critical features in a fluid and intermingled governance context. The identified critical features of (contemporary) mandated collaboratives may aid in assessing and improving interprofessional functioning within integrated elderly care. Intensifying (international) debate on governance expectations of mandated collaboratives may further contribute to sharpening the roles of both managers and healthcare professionals.

## Data Availability

The datasets generated and analysed during the current study are not publicly available as the informed consent applied only for the use by the research team. If desired, the data can be viewed and reviewed together with the corresponding author.
